# Psychosocial outcome of COVID-19 patients requiring ventilation after ECMO versus long-term mechanical ventilation

**DOI:** 10.3389/fcvm.2026.1709134

**Published:** 2026-02-24

**Authors:** I. Dalyanoglu, S. Seeger, L. J. Vallejo Castano, J. Nienhaus, E. Yilmaz, A. M. Markser, B. Korbmacher, A. Lichtenberg, H. Dalyanoglu

**Affiliations:** 1 Department for Vascular and Endovascular Surgery, Sankt Marien-Hospital Buer GmbH, Gelsenkirchen, Germany; 2 Department of Cardiac Surgery, University Hospital Duesseldorf, Duesseldorf, Germany; 3 Department of Anesthesiology, University Hospital Duesseldorf, Duesseldorf, Germany; 4 University Hospital Duesseldorf, Clinical Institute for Psychosomatic Medicine and Psychotherapy, Duesseldorf, Germany

**Keywords:** COVID-19, ECMO, ICU, mechanical ventilation, psychological outcome, PTSD

## Abstract

**Background:**

Severe COVID-19 frequently necessitates prolonged intensive care treatment, including long-term mechanical ventilation (MV) and extracorporeal membrane oxygenation (ECMO). While survival outcomes of these modalities have been extensively studied, data on long-term psychological sequelae remain limited. This study compared psychosocial outcomes in COVID-19 ICU survivors treated with ECMO vs. prolonged MV alone.

**Materials and methods:**

In this exploratory single-centre study combined retrospective clinical data with prospective long-term psychosocial follow-up, 150 adult patients with severe COVID-19 treated between March 2020 and December 2021 were included (ECMO: *n* = 98; MV: *n* = 52). Clinical data were collected retrospectively. The primary outcome of the study was long-term psychosocial outcome, which was assessed prospectively using validated questionnaires for depression, post-traumatic stress symptoms, attachment-related anxiety and avoidance, and health-related quality of life during structured long-term follow-up after ICU discharge.

**Results:**

ECMO patients were significantly younger (mean 53.8 vs. 66.0 years; *p* < 0.001) and required longer invasive ventilation (29.3 vs. 13.3 days; *p* = 0.011). Among survivors completing long-term follow-up, substantial psychological morbidity was observed in both treatment groups, with differences in attachment-related anxiety and numerically higher depressive and post-traumatic stress symptoms depending on ventilation strategy. Multivariate Cox regression identified older age, chronic obstructive pulmonary disease, and the need for hemodialysis as independent predictors of mortality A total of 29 survivors (ECMO: *n* = 16; MV: *n* = 13) completed psychological follow-up assessments. The observed pattern of higher depressive and trauma-related symptom burden among ECMO survivors may reflect the cumulative psychological impact of prolonged life-support, high perceived threat to life, and prolonged dependency during critical illness rather than a direct effect of ECMO itself. Survival did not differ significantly between groups.

**Conclusion:**

Survivors of severe COVID-19 requiring either ECMO or prolonged mechanical ventilation exhibit a substantial long-term psychological burden. Distinct psychosocial profiles were observed between treatment modalities, with higher attachment-related anxiety among MV survivors and numerically greater depressive and trauma-related symptoms among ECMO survivors. These findings highlight the importance of systematic post-ICU psychological screening and the integration of psychosocial outcomes into long-term critical care follow-up.

## Introduction

In December 2019, an outbreak of pneumonia of unknown origin emerged in Wuhan, China and was subsequently attributed to the novel severe acute respiratory syndrome coronavirus 2 (SARS-CoV-2), causing coronavirus disease 2019 (COVID-19) (1, 2). During the pandemic, a substantial proportion of patients developed severe respiratory failure requiring intensive care unit (ICU) treatment, including prolonged invasive mechanical ventilation (MV) and, in selected cases, extracorporeal membrane oxygenation (ECMO) for refractory acute respiratory distress syndrome (ARDS) (3–5).

ECMO represents a resource-intensive rescue therapy for patients with life-threatening respiratory failure. Veno-venous ECMO (VV-ECMO) is primarily used in isolated respiratory failure, whereas veno-arterial ECMO (VA-ECMO) is reserved for combined cardiac and pulmonary failure. During the COVID-19 pandemic, the use of ECMO increased markedly, particularly in patients with refractory hypoxemia despite optimized conventional ventilation strategies (6–8). While short-term survival and clinical outcomes of ECMO in COVID-19 have been extensively investigated, less attention has been paid to long-term patient-centered outcomes beyond survival.

Survivors of critical illness are known to be at high risk for persistent physical, cognitive, and psychological impairments, collectively referred to as post-intensive care syndrome (PICS). Psychological sequelae include symptoms of depression, anxiety, and post-traumatic stress disorder (PTSD), which may persist for months or years after ICU discharge (9–11). COVID-19 ICU survivors appear to be particularly vulnerable to long-term psychological morbidity. Potential contributing factors such as prolonged sedation, extended ventilation duration, delirium, and strict visitation restrictions have been described in the literature but were not systematically analysed in the present study and are therefore considered contextual factors rather than explanatory variables. Previous studies have reported clinically relevant PTSD symptoms in approximately 20%–30% of COVID-19 ICU survivors, as well as substantial rates of depression and impaired health-related quality of life (12, 13).

Beyond classical symptom-based assessments of depression and PTSD, individual vulnerability factors may influence long-term psychological outcomes after critical illness. Attachment theory provides a framework for understanding how individuals regulate stress and seek support under extreme conditions. Attachment-related anxiety and avoidance have been associated with increased psychological distress and reduced resilience in various medical and psychiatric populations. However, the role of adult attachment patterns in critically ill patients—particularly those undergoing highly invasive treatments such as ECMO—remains largely unexplored. The Experiences in Close Relationships-Revised questionnaire (ECR-R8) allows efficient assessment of attachment-related anxiety and avoidance and may provide complementary insights into psychosocial vulnerability after ICU treatment (14).

Given the high incidence of severe respiratory failure, prolonged mechanical ventilation, and frequent neuropsychological stressors in critically ill COVID-19 patients, there is a growing need to evaluate long-term outcomes that extend beyond survival. In particular, it remains unclear whether survivors treated with ECMO differ from those receiving prolonged mechanical ventilation alone with regard to psychological morbidity, attachment-related traits, and health-related quality of life. Therefore, the aim of this study was to compare long-term psychosocial outcomes in COVID-19 ICU survivors treated with ECMO vs. prolonged mechanical ventilation alone. Long-term psychosocial outcome was defined as the primary endpoint, while survival and other clinical variables were considered secondary outcomes. Specifically, we assessed symptoms of depression, post-traumatic stress, attachment-related anxiety and avoidance, and health-related quality of life during structured long-term follow-up after ICU discharge. These psychological outcomes were considered exploratory endpoints.

## Study design and methods

This single-centre, observational study was conducted at Düsseldorf University Hospital. The study population included patients with confirmed SARS-CoV-2 infections who were treated in the Department of Cardiac Surgery and received either prolonged mechanical ventilation (defined as ≥72 h) or additional extracorporeal membrane oxygenation (ECMO) therapy. The study incorporated both retrospective and prospective components Our study is thus a retrospective analysis of clinical data combined with prospective psychosocial follow-up.

A retrospective review of clinical records was performed for all ICU patients treated during the study period. Sources included discharge summaries, ICU charts, and laboratory data from the hospital's electronic records system. The primary endpoint of the study was long-term psychosocial outcome assessed during structured follow-up using validated psychological questionnaires (BDI-II, ITQ, SF-12, ECR-R8). Secondary endpoints included overall survival, duration of invasive mechanical ventilation, and baseline clinical characteristics derived from retrospective ICU data. To minimize selection bias, all consecutive ICU admissions meeting predefined inclusion criteria during the study period were included. Thus, the comparability of groups was assured by baseline characteristics (Table 1). The single-center design limits the generalizability of our findings, and selection bias is possible because only patients who survived and were available for follow-up could be assessed. Moreover, retrospective extraction of clinical variables did not allow for detailed assessment of important ICU exposures such as sedation depth, delirium episodes, family visitation, or the presence of ICU diaries, all of which may influence psychological outcomes. Detailed data on sedative regimens (specific agents, dosages, and depth of sedation) were not consistently available in the retrospective records and were therefore not included in the present analyses. Objective parameters of pulmonary disease severity at ICU admission, including CT severity scores, PaO_2_/FiO_2_ ratios, and levels of positive end-expiratory pressure (PEEP), were not consistently available in the retrospective records and could therefore not be included in the analyses.

**Table 1:** Clinical and demographic baseline characteristics of ECMO vs. MV patients.

Variable	ECMO^a^	MV^b^	*p*-value
Group size (*n*)	98	52	
VV-ECMO, *n* (%)^c^	83 (84.7%)		
VA-ECMO, *n* (%)^d^	15 (15.3%)		
Male sex	77	34	0.080
BMI^e^ (mean)	29.70	29.89	0.640
Age at admission (mean, in years)	53.77	66.02	<0.001*
Nicotine abuse	8	5	0.764
Hyperlipoproteinemia	10	13	0.017*
Arterial hypertension	48	38	0.005*
Coronary artery disease	27	16	0.678
Cerebrovascular disease	0	1	0.347
Peripheral artery disease	0	3	0.040*
COPD^f^	9	6	0.647
Hemodialysis	47	23	0.663
Tracheotomy	66	37	0.632
Duration of invasive ventilation in days (mean)	29.32	13.33	0.011*
Length of ICU^g^ stay in days (mean)	31.54	21.94	0.468

^a^ ECMO, extracorporeal membrane oxygenation.

^b^ MV, mechanical ventilation.

^c^ VV-ECMO, veno-venous ECMO.

^d^ VA-ECMO, veno-arterial ECMO.

^e^ BMI, body mass index.

^f^ COPD, chronic obstructive pulmonary disease.

^g^ ICU, intensive care unit.

* *p* ≤ 0,05; considered statistically significant.

**Figure 1:**
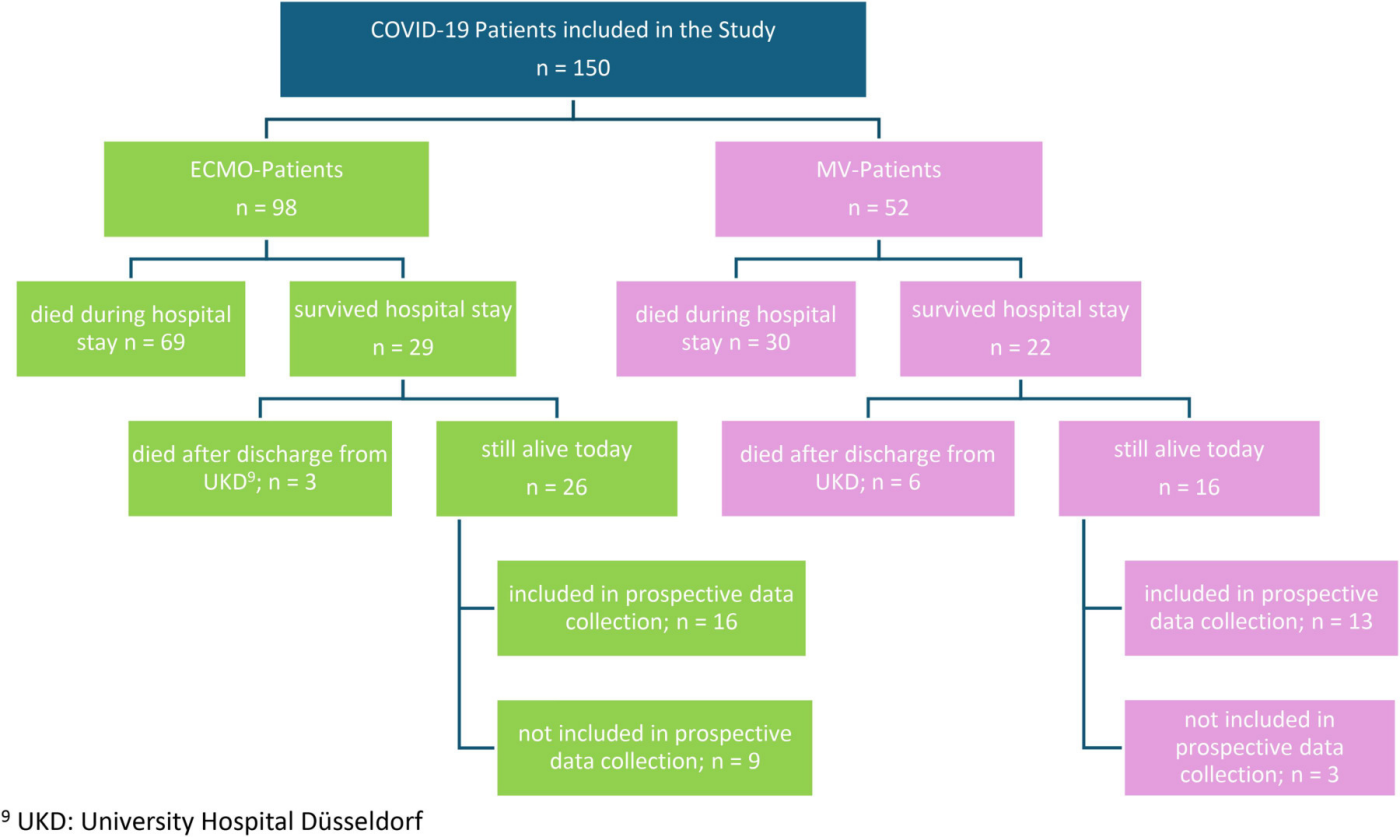
Patient flow and inclusion overview by treatment group (ECMO vs. MV). UKD, university hospital Düsseldorf.

In the prospective part of the study, surviving patients who could be contacted, provided consent, and were available for assessment underwent a psychological follow-up. Follow-up assessments were conducted at a median of 40 months after ICU discharge. This process entailed either a 30 min interview at the hospital or, if this was not feasible, a questionnaire sent via post or email. The patient flow and inclusion of patients in the study by treatment group is shown in Figure 1.

All psychological outcomes were assessed using the following validated and widely established instruments:

– The Beck Depression Inventory-II (BDI-II) was used to assess depressive symptoms. Consisting of 21 items, it provides a total score ranging from 0 to 63. According to the national care guidelines for unipolar depression, a score of 14 or more indicates a mild depressive syndrome. A score between 20 and 28 points indicates moderate depression, while a score of 29 points or more indicates severe depression (15). The BDI-II has excellent reliability and validity, including high internal consistency (Cronbach's α ≈ 0.91) and test-retest reliability (r = 0.93), as well as strong correlations with other depression measures (e.g., BDI-IA: r = 0.93; Hamilton Scale: r = 0.71) (16).
– The International Trauma Questionnaire (ITQ) assesses core symptoms of PTSD and complex PTSD based on the ICD-11 criteria (17). The ITQ comprises 18 items, with ratings ranging from 0 to 4. According to the ITQ scoring rules, items rated at least 2 (“moderate” or higher) were considered symptomatic. A PTSD diagnosis requires the endorsement of at least one symptom in each of the three PTSD clusters, as well as functional impairment. CPTSD also requires the endorsement of at least one symptom in each of the three clusters relating to disturbance of self-organization, as well as impairment. The ITQ demonstrates high internal consistency (α = 0.89–0.94) and comparable diagnostic performance to the PCL-5 (17).
– The SF-12 Health Survey measures physical (PCS) and mental (MCS) health-related quality of life. Two composite scores are provided: the Physical Component Summary (PCS) and the Mental Component Summary (MCS). Both scores are derived from weighted combinations of the 12 items. They are standardized to a mean of 50 and a standard deviation of 10 in the general population. Higher values indicate a better perceived level of physical or mental health. There is strong correlation with the extended version of the SF-36 (PCS: r = 0.951; MCS: r = 0.969), with good internal consistency (α = 0.76 for PCS, α = 0.89 for MCS) (18).
– The ECR-R8 (Experiences in Close Relationships-Revised) is an 8-item version of the ECR-RD used to assess attachment-related anxiety and avoidance. The mean score for each patient is calculated for both subscales. These can then be converted into T-scores using age- and sex-specific normative tables. Participants were instructed to answer the ECR-R8 items with regard to their current romantic partner; if no current partner was available, they were asked to refer to a most significant past romantic relationship. Individuals without any current or past romantic relationship were advised to leave the ECR-R8 items unanswered; in these cases, attachment scores were treated as missing data. This enables comparisons to be made both within groups and against the normative sample. Higher anxiety scores indicate greater attachment-related anxiety, while higher avoidance scores indicate greater attachment-related avoidance. The ECR-R8 correlates highly with the original version (anxiety: r = 0.92; avoidance: r = 0.90) and demonstrates good validity. Short forms with comparable content report internal consistency values around α = 0.87–0.88 (14, 19).

Patients were divided into two groups (ECMO and mechanically ventilated only) at a ratio of 1.9:1 (98 vs. 52 patients) (Table 1). Subgroup analyses were conducted based on body mass index (BMI) categorised as <30 vs. ≥30, sex. The primary clinical endpoints were overall survival and psychological outcome. All analyses are based on complete data at hospital discharge and first scheduled follow-up. Cases of loss to follow-up and missing data are acknowledged in the limitations section and were handled by complete-case analysis. Presence of delirium at any time point was recorded in the clinical documentation but was not analysed as a study endpoint due to the retrospective nature of ICU data and incomplete availability of standardized delirium assessments. Accordingly, no causal inferences regarding delirium and long-term psychosocial outcome can be drawn from the present dataset.

### Ethics

The study was approved by the local ethics committee (Heinrich Heine University Düsseldorf, Protocol #2023-2527, September 20, 2023) and conducted in accordance with the Declaration of Helsinki. Due to the retrospective and anonymized design, patient consent was waived.

### Statistical analysis

All statistical analyses were performed using IBM SPSS Statistics (Version 29.0.2.0). Kaplan–Meier analyses were created using R (Version R 4.5.1 GUI 1.82). Descriptive statistics were used to summarize demographic and clinical characteristics, including means, medians, standard deviations, and interquartile ranges for continuous variables, and frequencies and percentages for categorical variables. Group comparisons between ECMO and mechanically ventilated (MV) patients were conducted using Mann–Whitney U-tests for non-normally distributed continuous variables and chi-square tests for categorical variables. A significance level of *p* < 0.05 was considered statistically significant.

Kaplan–Meier survival analyses were applied to assess 30-day and overall survival differences between groups. Log-rank tests were used to evaluate statistical significance. To identify independent predictors of mortality, multivariate Cox proportional hazards regression was performed, including variables such as age, sex, presence of COPD, need for hemodialysis.

For psychological outcomes, scores from the BDI-II, ITQ, SF-12, and ECR-R8 questionnaires were analyzed as **exploratory endpoints** given the limited number of follow-up assessments. Given the limited number of follow-up assessments, correlation analyses between psychological measures (BDI-II, ITQ, SF-12, ECR-R8) were performed in the pooled cohort only; subgroup-specific correlations for ECMO and MV patients were not calculated to avoid unstable estimates and overfitting. Subgroup analyses were conducted based on sex, BMI category (<30 vs. ≥30). Where appropriate, multiple logistic regression models were used to explore associations between clinical variables.

All tests were two-sided, and adjustments for multiple comparisons were not applied due to the exploratory nature of the study. To improve interpretability, effect sizes and 95% confidence intervals were added. Multivariable modelling of psychological outcomes was not feasible due to the limited number of completed follow-up assessments and the resulting risk of model overfitting.

## Results

### Baseline characteristics and secondary clinical outcomes (survival and mortality predictors)

**Figure 2:**
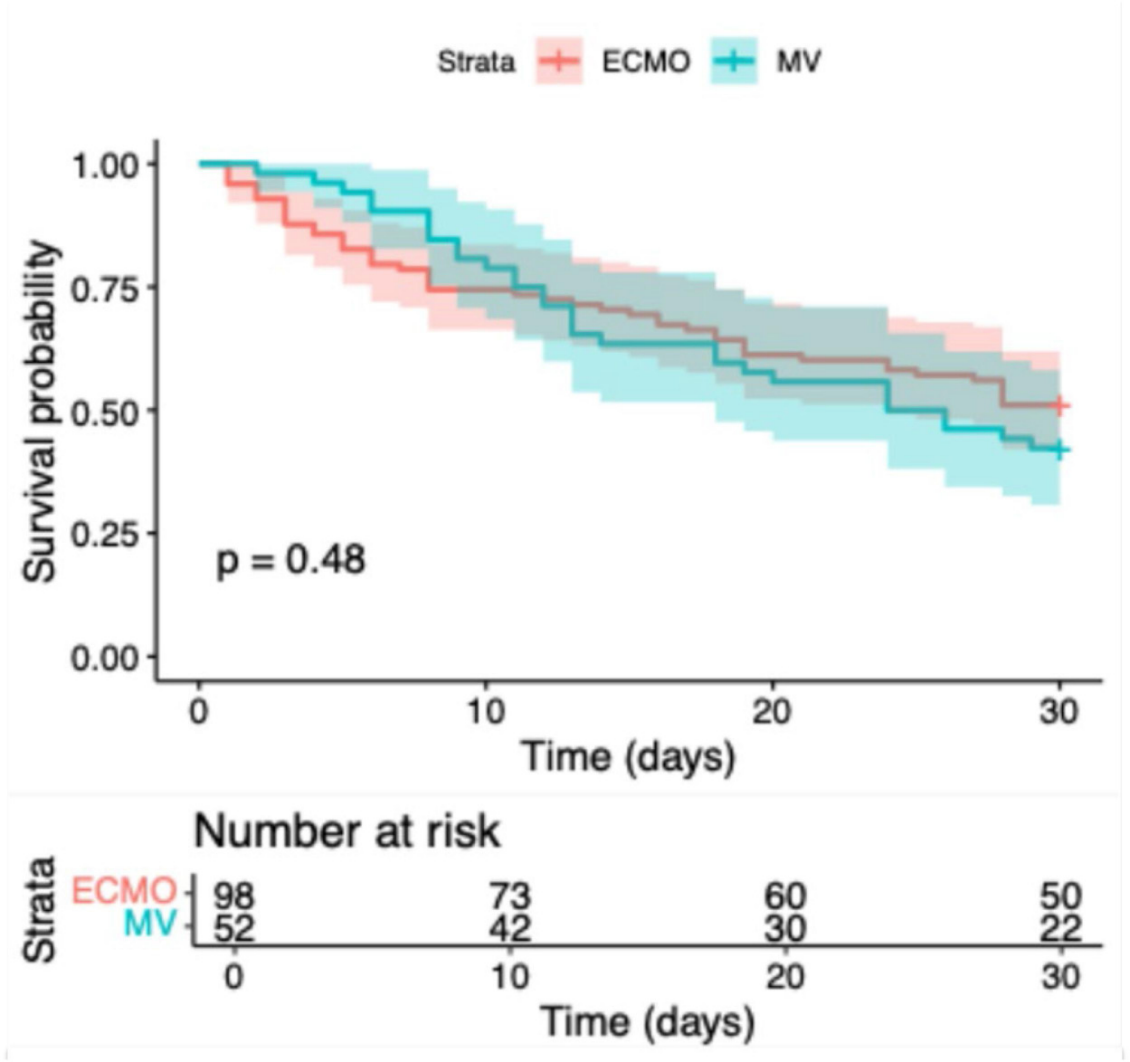
Kaplan–meier survival curve for 30-day in-hospital survival by treatment group (ECMO vs. mechanical ventilation) (*p* = 0.480).

**Figure 3:**
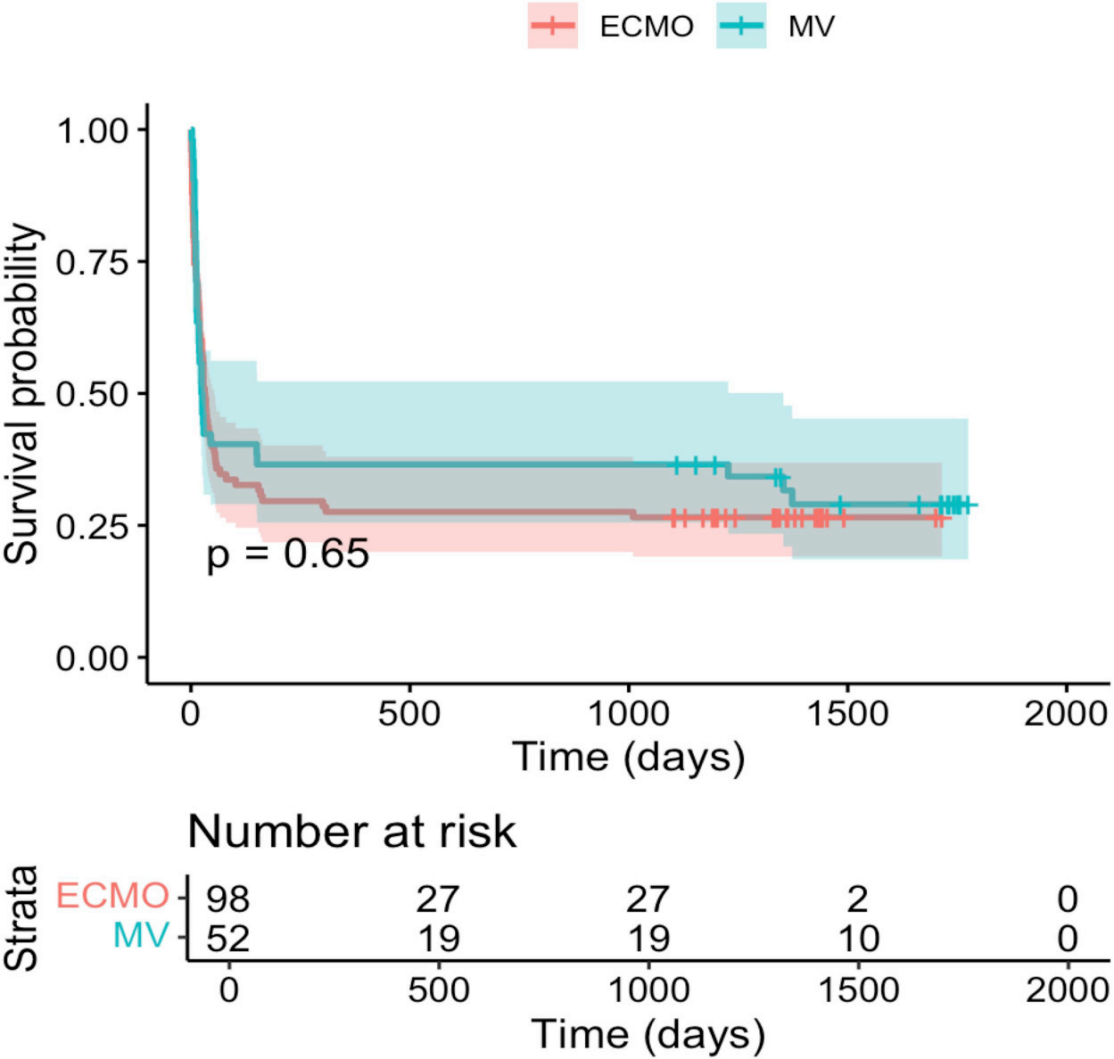
Kaplan–meier survival curve for overall survival by treatment group (ECMO vs. mechanical ventilation) (*p* = 0.649).

Baseline clinical and demographic characteristics of the study population are summarized in Table 1. Of the 150 included patients, 98 received extracorporeal membrane oxygenation (ECMO) and 52 were treated with prolonged mechanical ventilation (MV) alone. ECMO patients were significantly younger at ICU admission (mean age 53.77 vs. 66.02 years; *p* < 0.001) and required substantially longer durations of invasive mechanical ventilation (29.32 vs. 13.33 days; *p* = 0.011). Among ECMO patients, the majority were treated with veno-venous ECMO (*n* = 83 (84.7%), while a smaller proportion received veno-arterial ECMO for combined cardiopulmonary failure (proportions as shown in Table 1). The prevalence of most comorbidities was comparable between groups, although arterial hypertension and hyperlipoproteinemia were more frequent in the MV group. Body mass index and sex distribution did not differ significantly between groups. Kaplan–Meier survival analyses demonstrated no statistically significant differences between ECMO and MV patients with regard to 30-day in-hospital survival (log-rank *p* = 0.480; Figure 2) or overall survival (log-rank *p* = 0.649; Figure 3).

To identify independent predictors of mortality, a multivariate Cox proportional hazards regression analysis was performed (Table 2). Higher age at admission was associated with increased mortality risk [hazard ratio (HR) 1.039 per year; *p* < 0.001]. The presence of chronic obstructive pulmonary disease (COPD) (HR 2.107; *p* = 0.036) and the need for hemodialysis during ICU stay (HR 1.706; *p* = 0.023) were also independently associated with mortality. Duration of invasive mechanical ventilation was inversely associated with mortality (HR 0.989 per day; *p* = 0.045). Being conscious during ICU treatment was associated with lower mortality risk (HR 0.263; *p* < 0.001).

**Table 2:** Multivariate Cox regression: significant predictors of mortality in COVID-19 ICU patients.

Variable	Regression coefficient	SE (standard Error)	Significance (*p*-value)	Hazard Ratio
Age at admission (years)	0.038	0.010	<0.001*	1.039
COPD^a^ (yes = 1, no = 0)	0.745	0.356	0.036*	2.107
Hemodialysis yes = 1, no = 0)	0.534	0.235	0.023*	1.706
Duration of invasive ventilation (days)	−0.011	0.005	0.045*	0.989
Patient conscious (yes = 1, no = 0)	−1.337	0.288	<0.001*	0.263

^a^ COPD, chronic obstructive pulmonary disease.

* *p* ≤ 0,05; considered statistically significant.

### Psychological outcomes

Of all surviving patients, those who could be contacted, consented to participate, and completed the questionnaires were included in the psychosocial follow-up (Figure 1). Consequently, 29 survivors (ECMO *n* = 16, MV *n* = 13) formed the final cohort for psychological assessment, while non-responders and patients lost to follow-up were not analysed. Psychological follow-up assessments addressing the primary study endpoint (long-term psychosocial outcome) were completed by 29 survivors (ECMO: *n* = 16; MV: *n* = 13). These psychological outcomes represent exploratory endpoints due to the small sample size and high variability. Follow-up was conducted at a median of 35.10 months after ICU discharge in the ECMO group and 44.97 months in the MV group. Psychological outcome measures are summarized in Table 3. Depressive symptoms assessed by the Beck Depression Inventory-II (BDI-II) were numerically higher in ECMO survivors (mean 16.93; median 14) compared with MV survivors (mean 13.46; median 8), although this difference was not statistically significant (*p* = 0.406). Similarly, post-traumatic stress symptom severity measured by the International Trauma Questionnaire (ITQ) was higher in the ECMO group (mean 26.3; median 20) than in the MV group (mean 17.2; median 9; *p* = 0.332).

**Table 3:** Psychological assessment outcomes by treatment group (ECMO vs. mechanical ventilation).

Psychological assessment instruments	ECMO (mean)	ECMO (median)	MV (mean)	MV (median)	Significance Level (Mann–Whitney *U*-Test)
BDI-II^a^	16.93	14	13.46	8	0.406
ITQ^b^	26.3	20	17.2	9	0.332
SF-12^c^ PCS	38.92	34.9	44.20	45.2	0.420
SF-12^d^ MCS	40.38	39.6	48.53	46.6	0.160
ECR^e^-R8 avoidance (single scores)	2.62	2.50	2.56	1.88	0.187
ECR-R8 avoidance (T-scores)	51.77	52.00	49.67	47.00	0.189
ECR-R8 anxiety (single scores)	1.67	1.25	2.69	2.38	0.067
ECR-R8 anxiety (T-scores)	48.08	46.00	55.33	54.00	0.015*

^a^ BDI-II, beck depression inventory II.

^b^ ITQ, international trauma questionnaire.

^c^ SF-12 PCS, short form-12 health survey physical component summary.

^d^ SF-12 MCS, short form-12 health survey mental component summary.

^e^ ERC_R8, experiences in close relationships-revised.

* *p* ≤ 0,05; considered statistically significant.

Health-related quality of life assessed using the SF-12 showed lower physical component scores in ECMO survivors (mean 38.92) compared with MV survivors (mean 44.20; *p* = 0.420). Mental component scores were also lower in the ECMO group (mean 40.38 vs. 48.53; *p* = 0.160). These differences did not reach statistical significance. Attachment-related avoidance measured by the ECR-R8 showed no significant differences between groups, either for single scores (2.62 vs. 2.56; *p* = 0.187) or T-scores (51.77 vs. 49.67; *p* = 0.189). In contrast, attachment-related anxiety T-scores were significantly higher in MV survivors compared with ECMO survivors (55.33 vs. 48.08; *p* = 0.015). Differences in attachment-related anxiety single scores narrowly missed statistical significance (2.69 vs. 1.67; *p* = 0.067). Correlation analyses across the entire follow-up cohort demonstrated strong positive associations between depressive symptoms (BDI-II) and post-traumatic stress symptoms (ITQ) (*r* = 0.79, *p* < 0.001). Higher BDI-II scores were associated with higher attachment-related anxiety (*r* = 0.43, *p* = 0.032) and lower physical (SF-12 PCS; *r* = −0.53, *p* = 0.003) and mental health scores (SF-12 MCS; *r* = −0.72, *p* < 0.001). ITQ scores were correlated with attachment-related avoidance (*r* = 0.41, *p* = 0.041) and inversely correlated with physical (*r* = −0.59, *p* < 0.001) and mental health (*r* = −0.82, *p* < 0.001). Attachment-related avoidance was also inversely associated with mental health-related quality of life (*r* = −0.52, *p* = 0.008).

## Discussion

This single-center observational study investigated long-term psychosocial outcomes in survivors of severe COVID-19 requiring intensive care, comparing patients treated with extracorporeal membrane oxygenation (ECMO) to those receiving prolonged mechanical ventilation (MV) alone. The principal finding of this study is the presence of a substantial long-term psychosocial burden among survivors of severe COVID-19 requiring intensive care, regardless of whether ECMO or prolonged mechanical ventilation alone was applied. Differences in specific psychosocial dimensions, particularly attachment-related anxiety and trauma-related symptoms, suggest distinct vulnerability profiles associated with the respective treatment strategies. Taken together, the pattern of elevated depressive and post-traumatic stress symptoms, impaired SF-12 physical and mental health scores, and increased attachment-related anxiety in MV survivors is consistent with the multidimensional concept of post-intensive care syndrome (PICS).

In-hospital mortality in the present cohort was high, particularly among patients treated with ECMO. This finding likely reflects the selection of patients with more severe disease and refractory respiratory failure for ECMO support, as this therapy is typically reserved for the most critically ill individuals. Comparable observations have been reported in other ECMO cohorts, highlighting substantial inter-center variability related to patient selection and case severity (20). In the context of the relatively small sample size, overall mortality estimates may additionally be influenced by individual cases.

Kaplan–Meier analyses demonstrated no statistically significant differences in 30-day or overall survival between ECMO and mechanically ventilated patients in this cohort. ECMO was preferentially used in younger patients who were clinically assessed as having more severe respiratory failure, reflecting treatment selection rather than objectively quantified disease severity, as detailed physiological severity parameters were not available for analysis. Multivariate Cox regression analysis identified higher age at admission, the presence of chronic obstructive pulmonary disease, and the need for hemodialysis as independent predictors of mortality, underscoring the impact of comorbidity burden and multiorgan dysfunction on outcomes in critically ill COVID-19 patients. The inverse association between duration of invasive mechanical ventilation and mortality likely reflects survivor bias and should not be interpreted as a protective effect.

With regard to long-term psychological outcomes, survivors in both treatment groups demonstrated a substantial burden of depressive symptoms, post-traumatic stress symptoms, and impaired health-related quality of life several years after ICU discharge. From a conceptual perspective, both prolonged mechanical ventilation and ECMO represent extreme stressors that may exert long-term psychosocial effects through partially overlapping but distinct mechanisms. ECMO therapy is frequently associated with prolonged immobilisation, deep sedation, and an intense perception of life-threatening illness, which may facilitate trauma-related symptomatology. In contrast, prolonged mechanical ventilation without ECMO may be associated with longer periods of partial wakefulness, dyspnoea, and perceived helplessness, potentially affecting attachment-related anxiety and interpersonal security. These mechanisms are hypothesised based on established models of post-intensive care syndrome and attachment theory and should be interpreted as explanatory frameworks rather than causal conclusions.

ECMO survivors showed numerically higher levels of depression and PTSD symptoms, as well as lower physical and mental quality-of-life scores compared with patients treated with prolonged mechanical ventilation alone; however, these differences did not reach statistical significance. These findings are in line with previous studies reporting persistent psychological morbidity among ECMO and non-ECMO ARDS survivors following severe COVID-19 or other critical illnesses (20–25).

The lack of statistical significance should be interpreted in the context of the limited number of patients available for long-term psychological follow-up and the considerable interindividual variability in symptom severity. Consequently, the observed numerical differences may reflect clinically relevant trends that could not be confirmed statistically within the present cohort. Importantly, the high prevalence of depressive and trauma-related symptoms across both groups highlights the need for systematic psychological screening and structured post-ICU follow-up, regardless of the applied ventilation strategy.

### Comparison with non-COVID-19 ARDS populations

Existing literature on psychosocial outcomes in non-COVID-19 ARDS survivors requiring mechanical ventilation or ECMO suggests that the burden of depression, anxiety, and PTSD is substantial, even in the absence of SARS-CoV-2 infection (26, 27). Studies in influenza-associated ARDS and bacterial pneumonia-associated ARDS have reported PTSD prevalence rates between 20%–35% and significant long-term impairments in health-related quality of life, particularly in those requiring ECMO support. Several cohorts demonstrated no major difference in the pattern of psychological sequelae between COVID-19 and non-COVID ARDS survivors when controlling for ICU length of stay, sedation duration, and severity of illness (28). This indicates that while COVID-19 may amplify psychosocial distress due to isolation measures and visitation restrictions, the invasive nature of prolonged ventilation and ECMO itself remains a dominant factor in determining long-term mental health outcomes. The present findings are therefore consistent with the broader ARDS literature, underlining the need for structured post-ICU psychological screening and rehabilitation in both COVID- and non-COVID contexts.

### Extrapolation and external validity

Given the single-center design and the specific pandemic setting, the external validity and generalisability of our findings are limited. Patient management, ICU protocols, and psychosocial support structures may differ substantially between institutions and healthcare systems. Patient management, ICU protocols, and psychosocial support structures may differ substantially between institutions and healthcare systems; moreover, management strategies and psychosocial support have evolved since the early pandemic waves. Furthermore, the high incidence of isolation-related stressors during COVID-19 might not fully apply to non-pandemic ARDS cases. Nevertheless, the observed psychological patterns are consistent with pre-pandemic ARDS data, supporting cautious extrapolation.

### Group comparability and confounding

Although baseline demographic and clinical variables were largely comparable between ECMO and MV groups, statistically significant differences in age and duration of ventilation were observed. These differences likely reflect clinical decision-making and patient selection for ECMO, but they may also act as confounders for psychological outcomes. Although adjustment for major clinical covariates was performed, the possibility of residual confounding—common in observational ICU research—remains and should be explored in larger prospective datasets.

### Delirium as a predictor

In the present study, delirium was recorded but not analysed as a primary endpoint and should therefore be interpreted purely as a contextual factor for future hypothesis generation. Although delirium has been identified as a strong predictor of long-term psychiatric morbidity (12), our primary focus was on broader psychosocial outcomes. Given the limitations in sample size and follow-up data, a separate analysis for delirium was not feasible within the current dataset without compromising statistical validity; however, this remains a high-priority variable for future research. Larger cohorts should systematically evaluate delirium—including duration and severity—as a mediator between ICU treatment modality and long-term psychosocial health.

### Economic considerations

The cost estimates cited for ECMO and long-term PTSD/CPTSD care are based on existing literature and health system data; no primary cost analysis was conducted in this study. A formal economic evaluation was beyond our scope but is crucial for policy-making, given the substantial financial implications. Highlighting the integration of economic outcomes into post-ICU follow-up will be key for healthcare systems facing resource constraints post-pandemic.

Future work should incorporate prospective cost tracking and cost-utility analysis to better inform resource allocation. Beyond its life-saving potential, ECMO therapy is associated with considerable direct healthcare costs, frequently exceeding €50.000 per patient, excluding post-discharge rehabilitation and psychosocial care. From a public health perspective, the integration of mental health status, health-related quality of life, and functional recovery into cost-effectiveness analyses is essential for accurate resource planning. The economic considerations discussed here are based on previously published cost estimates; no primary cost analysis was performed in our cohort and these remarks are intended to guide future research rather than to support specific policy conclusions.

### Statistical power

The lack of statistically significant differences in most psychological outcomes, despite notable numerical trends, raises the possibility of a Type II error. Our sample size was limited by the strict inclusion criteria and the high in-hospital mortality of COVID-19 ECMO patients, which reduced the number of survivors available for follow-up. As such, subtle but clinically meaningful differences may exist and warrant confirmation in larger, multicenter follow-up studies. Larger multicenter studies with standardized follow-up intervals are needed to confirm these patterns.

## Clinical implications and future directions

Our findings underscore that severe COVID-19 ICU treatment, regardless of whether patients receive ECMO or prolonged mechanical ventilation alone, is associated with substantial long-term psychological morbidity and therefore requires structured post-ICU mental health screening and targeted support for all survivors. In our cohort, ECMO survivors tended toward numerically higher depression (mean BDI-II 16.9 vs. 13.5) and PTSD scores (mean ITQ 26.3 vs. 17.2), while mechanically ventilated patients showed significantly higher attachment-related anxiety T-scores (mean 55.3 vs. 48.1, *p* = 0.015). These findings indicate distinct mental health risk profiles that may require tailored, modality-specific interventions, including attachment-informed care for prolonged MV survivors and should be interpreted as exploratory, given multiple unadjusted comparisons and small sample size. Potential measures include systematic post-ICU mental health screening, trauma-informed rehabilitation programmes that address both physical recovery and psychosocial reintegration, early referral to specialized psychological care, and structured follow-up clinics that incorporate social support and targeted interventions for attachment-related difficulties. Future research should investigate mechanisms driving these differences—such as sedation protocols, delirium incidence, socioeconomic/demographic and biographical variables, or patient expectations—and test targeted interventions in specific subgroups.

The identification of modifiable clinical risk factors, such as the association of hemodialysis with increased mortality, underscores the importance of early organ-protective strategies and supports a nuanced approach to ECMO decision-making that explicitly weighs survival prospects against potential long-term psychosocial sequelae and resource constraints. Renal-preserving approaches may not only improve survival but could also reduce the long-term psychological burden linked to critical illness.

To confirm and expand these findings, larger multicenter studies with longitudinal follow-up are essential. Such studies should include both COVID-19 and non-COVID ARDS populations to evaluate generalisability and prospectively incorporate cost-effectiveness and health economic analyses to inform policy-making and optimize the allocation of critical care resources. On the health system level, these data argue for proactive service planning, including early mobilization strategies, ICU diaries, structured family engagement where feasible, and dedicated post-ICU follow-up services to accommodate the anticipated rise in mental health care needs among ICU survivors. Subsequent investigations should prioritize multicenter, prospective cohorts with standardized timing of psychological assessments, systematic collection of pre-ICU psychiatric baseline data, and detailed recording of ICU process variables (including delirium occurrence and duration, sedation regimens, family contact, and rehabilitation intensity) to allow robust multivariable adjustment for these potential confounders.Longitudinal designs are needed to delineate trajectories of depression, PTSD, and attachment-related anxiety over time and to identify patient subgroups who benefit most from specific interventions. In addition, randomized or quasi-experimental trials of psychosocial strategies, such as ICU diaries, structured family engagement, and early trauma-focused psychotherapy, are essential to determine their effectiveness in reducing long-term psychological morbidity after ICU care. Future cost-effectiveness analyses should explicitly incorporate psychosocial outcomes, health-related quality of life, and long-term functional recovery when comparing ECMO with alternative treatment strategies, in order to inform evidence-based resource allocation and critical care policy.

## Limitations

This study has several limitations that must be acknowledged.

– It is limited by its single-center design and relatively small sample size. All patients were treated within a single specialized ICU, which limits external validity. Differences in patient management and psychosocial support between pandemic and non-pandemic settings further restrict generalisability. Given the small follow-up sample and the exploratory design, all psychological findings—including the higher attachment-related anxiety in MV survivors—should be regarded as hypothesis-generating rather than confirmatory.
– The retrospective nature of clinical data and potential selection bias in the prospective follow-up are acknowledged. Specifically, the psychosocial analysis is restricted to survivors who were contactable and willing to participate several years after ICU discharge, which entails a considerable risk of selection and response bias. It is therefore possible that patients with more severe psychological morbidity are under- or overrepresented in the follow-up cohort, limiting the generalisability of the reported prevalence estimates and between-group comparisons. No pre-ICU psychiatric baseline data were available, which limits causal inference and precludes differentiation between pre-existing and new-onset psychiatric morbidity. Moreover, key ICU process variables (e.g., delirium occurrence and duration, sedation strategies, family visitation practices, and pandemic-specific stressors such as isolation or bereavement) were not systematically recorded and could not be incorporated as confounders into the analyses. In the absence of randomization, treatment allocation was based on clinical judgment, introducing the possibility that observed outcome differences reflect baseline severity rather than treatment effects. Furthermore, no standardized information on sedative medications and sedation depth was available across the entire cohort, precluding any meaningful comparison of sedation strategies between ECMO and MV patients and limiting our ability to assess their impact on long-term psychological outcomes.
– Kaplan–Meier survival analysis did not adjust for illness severity and may be influenced by unmeasured confounders; future studies should apply multi-variable adjusted survival analysis to improve the accuracy of outcome comparisons.
– Only one statistically significant difference was detected among multiple psychological endpoints, which, in the context of small and unequal follow-up groups, likely reflects limited statistical power and a considerable risk of Type II error; as a result, clinically relevant differences may have remained undetected. Furthermore, given the exploratory design and the absence of formal adjustment for multiple comparisons across psychological endpoints, the isolated statistically significant finding regarding attachment-related anxiety should be interpreted with caution
– No systematic primary data on post-discharge rehabilitation or economic costs were collected; cost estimates were derived from published data. Future studies should include prospective cost tracking.

## Conclusion

ECMO survivors showed numerically higher depression (BDI-II 16.9 vs. 13.5) and PTSD scores (ITQ 26.3 vs. 17.2), while mechanically ventilated survivors had significantly higher attachment-related anxiety T-scores (55.3 vs. 48.1, *p* = 0.015). These exploratory findings suggest potentially distinct mental health risk profiles that warrant confirmation in larger cohorts and should not be interpreted as definitive group differences given the limited sample size and lack of statistical significance for most psychological endpoints. Despite these exploratory findings regarding the primary psychosocial outcome, survival did not differ significantly between groups (30-day: *p* = 0.480; overall: *p* = 0.649).

These results highlight substantial long-term psychological burden across both treatment groups and underscore the need for systematic post-ICU mental health screening. Given comparable survival, high ECMO costs (>€50,000/patient), and persistent psychosocial morbidity, future studies should evaluate psychosocial outcomes alongside traditional endpoints when assessing ECMO utility.

Similar patterns in non-COVID ARDS survivors suggest invasive ventilation/ECMO—rather than COVID-19 specifically—drives long-term mental health sequelae.

## Data Availability

The raw data supporting the conclusions of this article will be made available by the authors, without undue reservation.
